# Protogynous functional hermaphroditism in the North American annual killifish, *Millerichthys robustus*

**DOI:** 10.1038/s41598-022-12947-2

**Published:** 2022-06-02

**Authors:** Omar Domínguez-Castanedo, Sharon Valdez-Carbajal, Tessy M. Muñoz-Campos, Jean H. Huber, Martin Reichard

**Affiliations:** 1 Asociación Mexicana para el Estudio y Conservación de Cyprinodontiformes, Tlalpan, C.P. 14390 CDMX, Mexico; 2 Licenciatura en Biología, Universidad Autónoma Metropolitana, Unidad Xochimilco, Calzada de Hueso 1100, Coyoacán, CDMX Mexico; 3 Ichtyologie, Muséum national d’Histoire naturelle, 53 rue Cuvier, 75005 Paris, France; 4 Institute of Vertebrate Biology, Czech Academy of Sciences, Brno, Czech Republic; 5 Department of Ecology and Vertebrate Zoology, Faculty of Biology and Environmental Protection, University of Łódź, Łódź, Poland; 6 Department of Botany and Zoology, Faculty of Science, Masaryk University, Brno, Czech Republic

**Keywords:** Evolutionary ecology, Wetlands ecology, Ichthyology

## Abstract

Sex change (sequential hermaphroditism) has evolved repeatedly in teleost fishes when demographic conditions mediate fundamentally different sex-specific returns for individuals of particular age and size. We investigated the conditions for potential sex change in an annual killifish (*Millerichthys robustus*) from temporary pools in Mexico. In natural populations, we detected adults with intersex colouration and gonads. Therefore, we experimentally tested whether this apparent sex change can be generated by manipulation of ecological and social conditions, rather than being caused by environmental disturbance. We demonstrated functional protogynous (female-to-male) sex change in 60% replicates, when groups of five females interacted and had a visual and olfactory cue of a male. Only one female changed sex in any given replicate. The sex change never occurred in isolated females. Protandrous (male-to-female) hermaphroditism was not recorded. We characterized gradual changes in behaviour, colouration and gonad structure during the sex change process. The first behavioural signs of sex change were observed after 23 days. Secondary males spawned successfully after 75 days. We discuss the adaptive potential of sex change in short-lived annual fishes through the seasonal decline of males, and during colonization of new habitats. This is the first observation of functional hermaphroditism in an annual killifish.

## Introduction

Sequential hermaphroditism (sex change) is taxonomically widespread among animals, but within vertebrates, it is unique to teleost fishes^1,2^. It is a highly complex phenomenon of evolved individual plasticity governed by changes in social and ecological factors^3^. Some fishes reproduce first as males and change to highly fecund females (protandry). Other fishes reproduce first as females and change to large dominant males (protogyny). Bi-directional sex-change also exists, whereby individuals may switch between being functional males and females^4,5^. Such plasticity is conserved within species but may be highly variable within lineages of closely related species^6^.

It was originally proposed that sequential hermaphroditism can evolve as a mechanism of population density regulation, in which individuals of certain age classes change their sex to compensate for sex ratio biases in very sparse or dense populations^7–9^ or to increase the total zygotic production of a population^10^. Recent models suggest a crucial role of selection operating differentially on individual fitness to induce the sex change, acting through life history strategy to maximize individual fitness in a particular socioecological context^3,11^. Consequently, there is clear behavioural control over sex change, where social or mating systems confer differential advantages for each sex under particular social and ecological conditions^1^. We now have a solid mechanistic understanding of sex change, including hormonal, histological and molecular mechanisms of the process^12^, especially in sparid, gobiid and serranid fishes where sequential hermaphroditism is common^13^. These fishes are typically marine, long-lived and may form relatively compact social groups.

Cyprinodontiform fishes (order Cyprinodontiformes) are small fishes, colloquially called killifishes and livebearers, with many species expressing peculiar life histories. While Poeciliidae are mainly ovoviviparous and Goodeinae contain strictly viviparous species, most cyprinodontiforms are oviparous killifishes (e.g. Nothobranchiidae, Fundulidae, Rivulidae, Procatopodidae). Cyprinodontiform fishes are popular among hobbyists and sex change has occasionally been reported anecdotally, especially in Poecilidae. For the ovoviviparous swordtail *Xiphophorus helleri* (Poeciliidae), there is experimental evidence of protogynous sex change of a laboratory strain, supported by histology^14^. Two case accounts of aged wild non-annual oviparous killifish *Aaptichilichthys websteri* (Procatopodidae) and *Pseudepiplatys annulatus* (Nothobranchiidae) reported that one of the functional females changed phenotypically to a male^15,16^, though their gonad histology was not examined.

Annual life history, whereby fish inhabit temporary water bodies, has evolved repeatedly in oviparous cyprinodontiforms, at least twice in African Nothobranchiidae and four times in Neotropical Rivulidae^17,18^. The life cycle of annual killifish is condensed into a single year due to the seasonal desiccation of their habitats^19^. Populations persist through the drought in the form of embryos arrested in diapause and buried in the dry substrate until the next rainy season^20–23^. When temporary pools are filled with rainwater the embryos hatch, grow fast and mature early to produce the next generation of drought-resistant embryos before the seasonal drought. Over their adult stage, the proportion of males in the population often substantially decreases^24,25^ due to the combined effects of sex-specific predation on the more colourful and ornamented males^26,27^ and higher background male mortality^28^. This may lead to extremely biased sex ratios in natural populations. In African *Nothobranchius furzeri*, sex-bias in some populations may reach < 10% of males^24,28^ and the proportion of males gradually decreases over the season in African^29^ and Neotropical annual killifish^30^. Under these circumstances, the potential for protogynous sex change appears adaptive.

**Figure 1:**
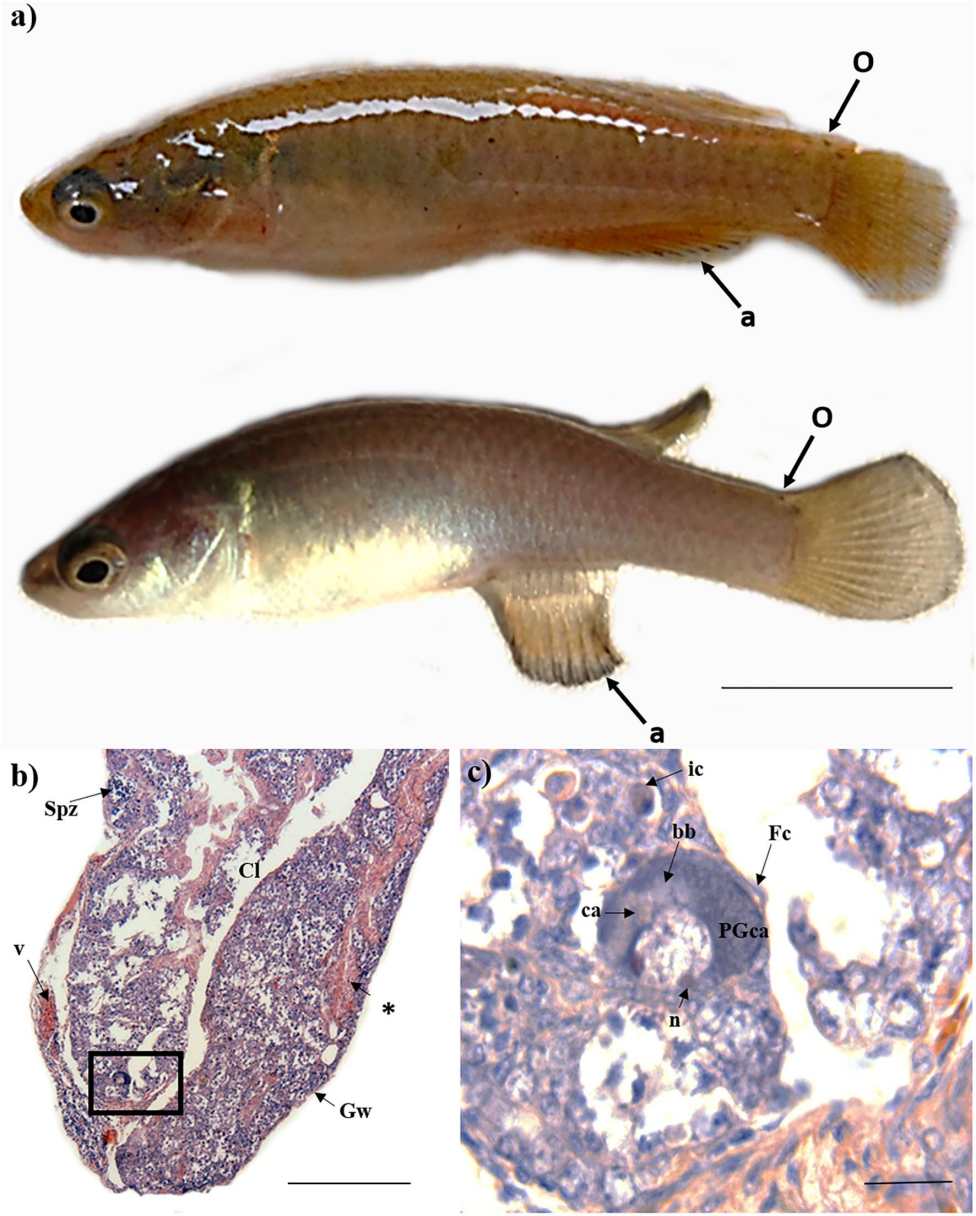
Intersexual *Millerichthys robustus* individuals collected in temporary pools during the flood phase in Veracruz, México. (**a**) Fish with phenotypic characteristics of a female (ocelli in the base of the caudal fin [o]) and a male (three-colour pattern in the anal fin [a]). The fish at the top was sampled in "Jesús Carranza" (N17° 26′ 39.16″, W95° 1′ 27.90″) during the fall of 2015; and the lower fish was collected near of Tlacotalpan (N 18° 37′ 06.08″, W95° 38′ 08.71″) during the winter of 2016. Scale bar = 10 mm. (**b**) Panoramic view of a longitudinal section of intersexual gonad [HE staining] with sperm cells (Spz) within the cellular cysts, and ovarian follicles. Irregular lamellae are seen projecting from the wall of the gonad (Gw) into the central lumen (Cl). Blood vessels (v) and efferent duct-like structures (*) are seen. Scale bar = 100 µm. (**c**) Detail of the ovarian follicle in the Cortical alveoli stage (PGca) [HE staining]. In the cytoplasm, the Balbiani body (bb) with acidophilic cortical alveoli (ca) is observed. In the nucleus, there are nucleoli (n) arranged in contact with the inner nuclear membrane. In addition, follicular cells (Fc) are observed in the follicular periphery. Multiple immune system (ic) cells are seen in the gonad lamellae. Scale bar = 10 µm.

The family Rivulidae is species-rich clade of killifish, which is distributed in the Neotropics. Several lineages have evolved extreme life-history strategies to survive in highly complex ephemeral ecosystems, such as breeding out of the water, pseudo-ovoviviparity, delayed hatching, production of desiccation-resistant embryos, simultaneous hermaphroditism with self-fertilization, and annual life history through embryonic diapause^31,32^. *Millerichthys robustus* is the only known annual killifish in North America, endemic to the basins formed by the Coatzacoalcos and Papaloapan rivers, in Veracruz and Oaxaca, Mexico^33,34^. Extensive field sampling^17,35–37^ indicated the possible occurrence of hermaphroditism in this annual fish. Namely, we detected (i) that some adult males (in various wild populations) possessed small ocelli on the upper base of the caudal fin, which is otherwise a phenotypic characteristic of female colouration (Fig. 1a); and (ii) the existence of individuals with female secondary sexual characteristics, whose gonads contained a mix of testicular and ovarian tissues (Fig. 1b,c). However, it remains to be tested whether this apparent sex change is modulated by ecological and social conditions, because hermaphroditism and intersexual fish can be triggered by environmental challenges such as water pollution^38^.

Here, we experimentally test (i) whether the observation of intersexual individuals in *M. robustus* pertains to functional hermaphroditism, (ii) the direction of the sex change and (iii) the social conditions associated with the capacity to change the sex. We compared behavioural, phenotypic (colouration) and histological traits associated with sex change in an interval of 90 days, after which the initiated sex change was fully complete.

## Results

To test whether mate competition induces sex change in *M. robustus*, we divided replicated experimental tanks into three experimental compartments using mesh screens that allowed visual and chemical interaction but prevented physical contact. Protogynous (female-to-male) sex change was recorded in 9 of 15 (60%) replicates in the treatment where the group of five females physically interacted. In each of the 9 replicates, only one female in the tank changed her sex. In the isolated female treatment, no sex change occurred (n = 15). Protandrous (male-to-female) sex change was never observed in any treatment (n = 15 for interacting males and n = 15 for isolated males). The first (behavioural) signs of protogynous sex change were observed 23 days after treatment commencement (median 33 days, range 23–41 days, n = 9).

When we compared female behaviour prior to sex change (Days 1–21) between replicates when the sex change did subsequently occur and those with no sex change, we found no difference in the rates of harassment (GLMM with negative binomial error, z = − 0.59, P = 0.554, n = 165), opercular expansion (GLMM with Poisson error, z = 0.52, P = 0.604, n = 165), frontal displays (GLMM with Poisson error, z = -0.05, P = 0.963, n = 165) or attacks (GLMM with Poisson error, z = 0.84, P = 0.404, n = 165). Females changed their sex irrespective of the spatial arrangement of the experimental compartments. Specifically, sex change occurred when the group of females was positioned in the middle (between compartment with a pair and compartment with single female, in 3 of 5 cases) or at any side [adjacent to either only a reproducing pair (2 of 5) or only a single female (4 of 5)].

As protogynous sex change occurred strictly in treatments with female-female physical interactions, the dynamics in behavioural (defined in Table 1) and phenotypic traits is described only in this treatment. The fish displayed typical female-female agonistic interactions at the beginning of the experiment (prior to sex change and in its initial stages). In replicates where sex change was recorded, we observed a decrease in female aggression behaviours and increase in reproduction-associated behaviour.

**Table 1:** Behavioral units of *Millerichthys robustus*. Description, mode of use and sex-specificity of particular behaviours are shown. Modified from Valdesalici et al.^59^ and Domínguez-Castanedo^40^.

Behaviour	Description	Expression	Sex specificity
Harassment	Fish slowly approaches the opponent frontally and keeps at a distance that avoids physical contact with the opponent. It is generally directed towards the anterior region of the opponent	Agonistic interactions	Male and female towards same-sex individual
Attack	Fish swims towards the opponent and bites or attempts to bite its fins or body	Agonistic interactions or reproductive behaviour	Male and female towards same-sex individual. Sometimes males towards females
Operculum expansion	Fish spreads the operculum and mouth and swims frontally, focused on the frontal region of the opponent	Agonistic interactions	Male and female towards the same-sex individual
Frontal display	Body moves in sigmoid undulations, fish swims frontally towards the opponent. Fins fold and unfold based on sigmoidal motion	Agonistic interactions	Male and female towards same-sex individual
Lateral display	Fish directs its flank parallel to the opponent’s flank and maintains the position	Agonistic interactions or reproductive behaviour	Male towards another male or females
Sigmoid display	Fish deploys sigmoid display flush against the substrate, alternating it with body undulations	Reproductive behaviour	Males to females
Dive	Fish dive into the subtrate to spawn following the female	Reproductive behaviour	Males to females
Spawning	The fish under the substrate spawn	Reproductive behaviour	Males with females

**Figure 2:**
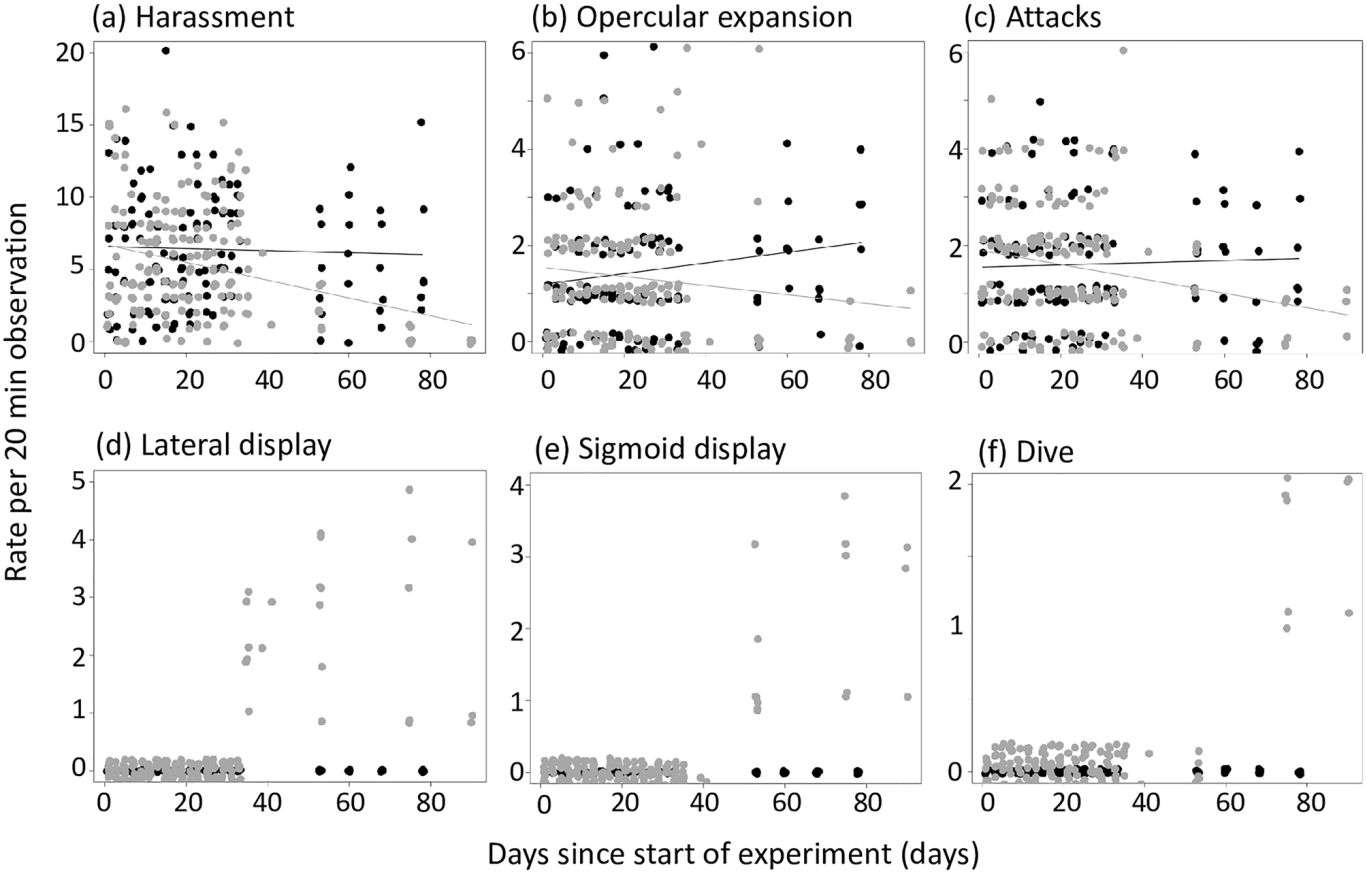
Rate of behaviours (as defined in Table 1) over the course of the experiment in replicates with (grey marks) and without (black marks) sex change, with trend lines. Data points are jittered to improve visibility.

Specifically, the rate of three of four agonistic behaviours declined in replicates where sex change had occurred, but not in those where all individuals remained functional females, as revealed by significant time by sex change interaction terms in the Generalized Linear Mixed Models (GLMMs) with negative binomial distribution (n = 298). Those behaviours were harassment (p = 0.001), opercular expansion (p = 0.006) and attacks (p = 0.014) (Table 2, Fig. 2). A similar decline was observed in frontal displays, but the difference between replicates with and without sex change was not statistically significant (GLMM, p = 0.115; Table 2).

**Table 2:** The effects of time (in days), the occurrence of sex change and their interaction on the expression of harassment (a), opercular expansion (b), frontal displays (d) and attacks (d). Fitted error distribution (in parentheses), parameter estimates and z-statistics of Generalized Mixed Models are presented.

Behaviour	Factor	Estimate	± SE	z-value	P
Harassment (nbinom2)	Intercept	1.881	± 0.104	18.13	< 0.001
	Days	− 0.001	± 0.003	− 0.39	0.697
	Sex change(males)	0.150	± 0.143	1.05	0.293
	Days × sex change	− 0.017	± 0.005	− 3.42	0.001
Opercular expansion (nbinom1)	Intercept	0.196	± 0.141	1.39	0.164
	Days	0.007	± 0.003	2.05	0.040
	Sex change(males)	0.266	± 0.187	1.43	0.154
	Days × sex change	− 0.016	± 0.006	− 2.78	0.006
Frontal display (nbinom2)	Intercept	0.651	± 0.112	5.83	< 0.001
	Days	0.0001	± 0.003	0.118	0.906
	Sex change(males)	0.153	± 0.149	0.91	0.364
	Days × sex change	− 0.008	± 0.005	− 1.57	0.115
Attacks (Poisson)	Intercept	0.437	± 0.117	3.75	< 0.001
	Days	0.001	± 0.003	0.43	0.669
	Sex change(males)	0.245	± 0.154	1.59	0.113
	Days × sex change	− 0.013	± 0.005	2.45	0.014

**Figure 3:**
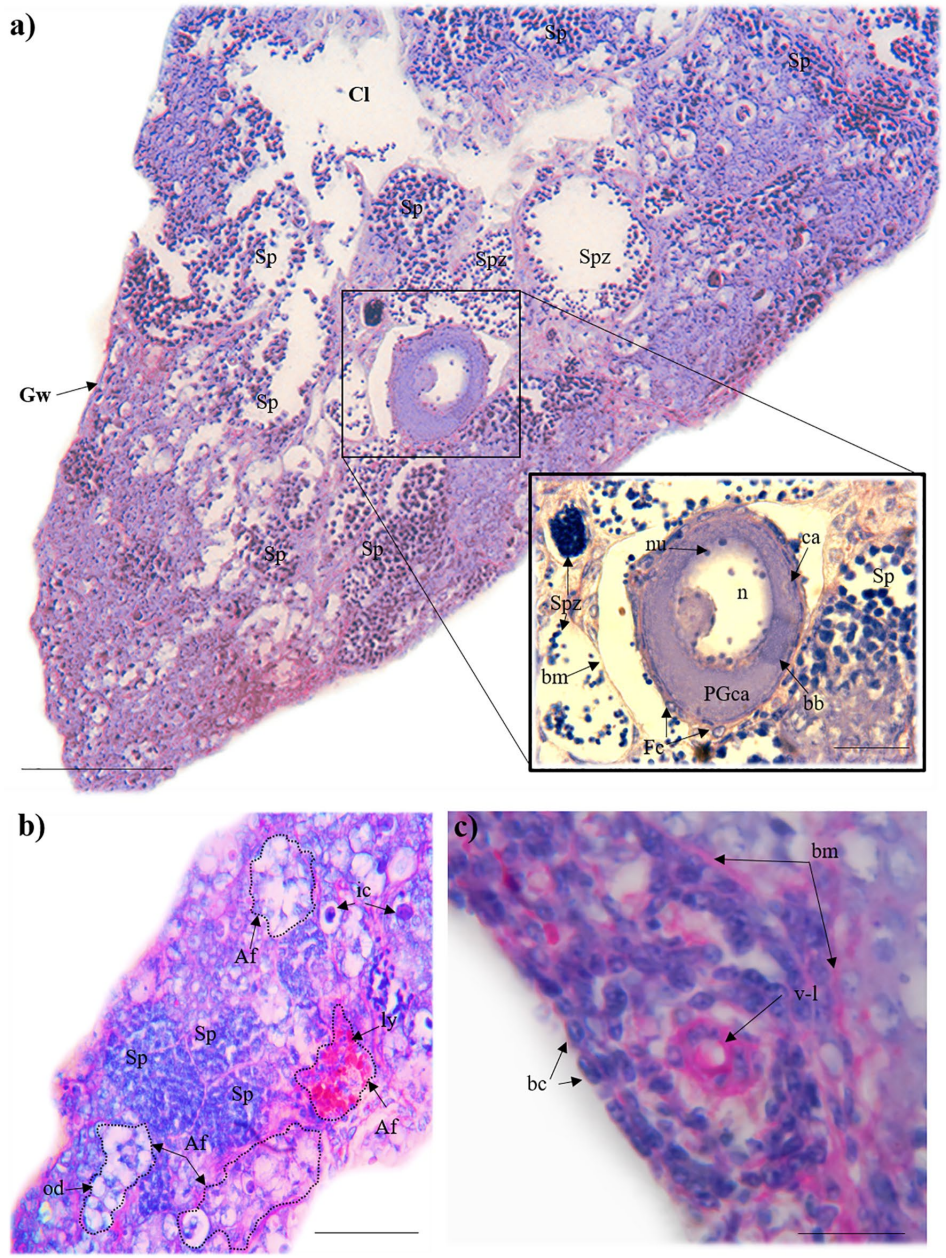
Histology of the intersexual gonads of *Millerichthys robustus* at 35 ± 8 days post start of the experiment [PAS staining]. (**a**) Panoramic longitudinal section of intersexual gonad [PAS staining] with numerous cysts containing spermatozoa (Spz) and primary spermatocytes (Sp) in the lamellae projecting from the walls of the gonad (Gw) to the central lumen (Cl). Scale bar = 100 µm. In detailed segment, an ovarian follicle is shown in the passage of the cortical alveoli (PGca) of the primary growth. Multiple nucleoli (nu) are observed in the periphery of the nucleus, cortical alveoli (ca) scattered in the cytoplasm, and the body of Balbiani (bb) arranged in a region peripheral to the nucleus (n). In addition, intact follicular cells (Fc) are observed in the periphery of the follicle. Spermatozoa (Spz) and primary spermatozoa (Sp) surrounded by a basement membrane (bm) are also observed. Scale bar = 25 µm. (**b**) Lamella with atretic ovarian follicles in different absorption states (Af) indicated in dotted lines, primary spermatocytes (Sp), and cells of the immune system (ic). Note that drops of fat (od) and liquefaction yolk (ly) can be seen in the atretic follicles. Scale bar = 45 µm. (**c**) Detail vessel-like structures (v-l) associated with basophile cells, near a basement membrane and atretic follicles (bm). Scale bar = 10 µm.

**Figure 4:**
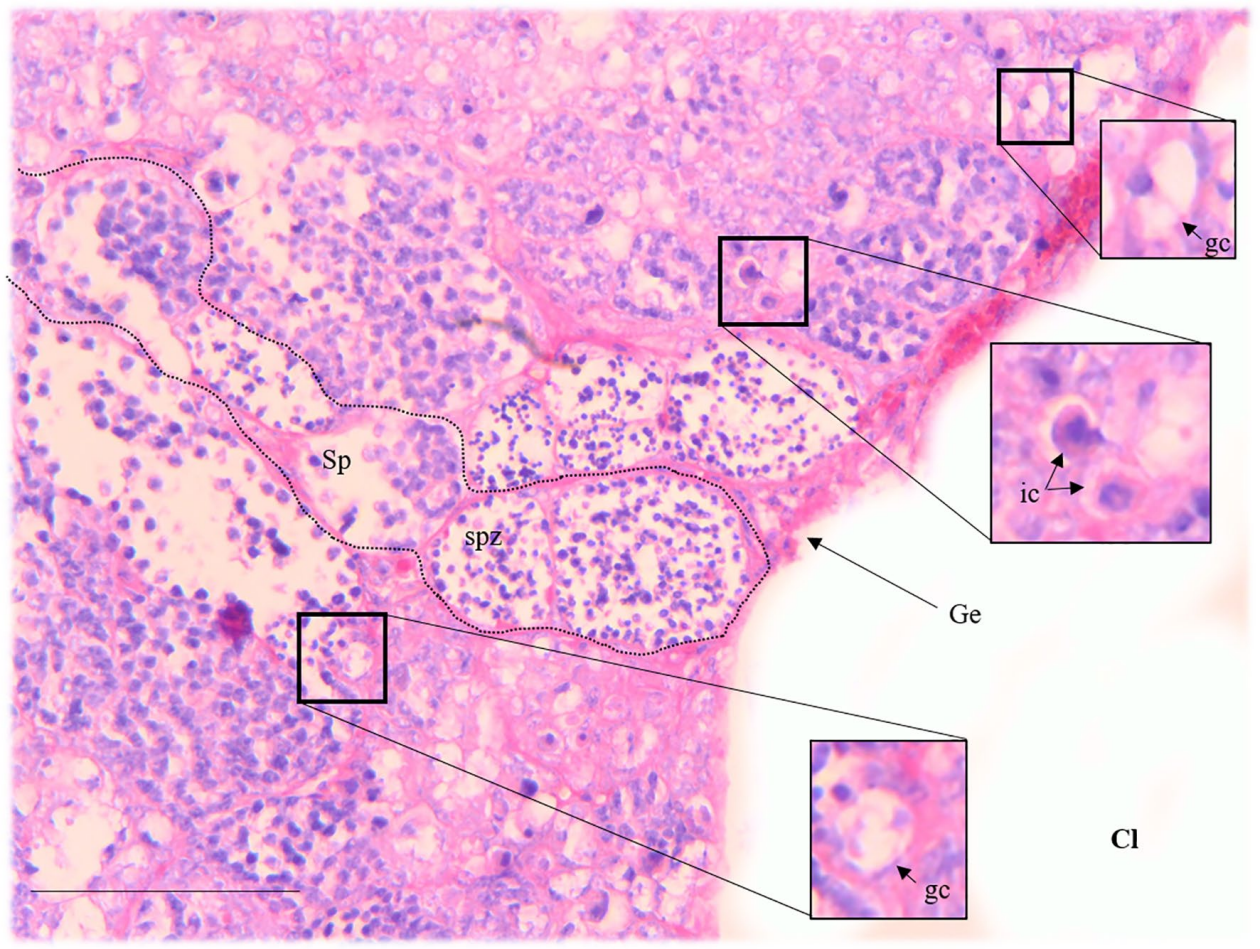
*Millerichthys robustus* gonad with testicle-like structure [HE staining]. Transversal section of gonads with the lobular structure of the testis, with spermatozoa (spz), and primary spermatocytes (Sp). In the same section, sections with disarranged cellular material, with immune system cells (ic) and germ cells (gc) with hyaline cytoplasm and prominent basophile nucleoli (gc) can be observed. Scale bar = 25 µm.

The first typical male behaviour was observed at Days 23–41, when no male colouration was apparent. This behaviour, lateral displays, is typical male behaviour used in both aggressive interactions and courtship, and occurred at a rate of 2.5 ± 0.24 per 20 min (mean ± s.e.), with no temporal trend between days 35 and 90. Histological analysis of the gonad tissue at this time revealed the existence of intersexual gonads, in which ovarian and testicular tissues co-occurred (Fig. 3a). This appeared to be a sequential process and it was infrequent to observe the simultaneous presence of female and male gametes. Instead, we commonly observed degenerating female gametes with developing male germ cells and rearrangement of the interstitial tissue (Fig. 3b). Numerous small basophilic cells near to the atretic follicles were observed, near to vessels-like structures (Fig. 3c). Interestingly, even in the most advanced follicles in atresia, the rupture of follicular basement membrane was not observed. A central lumen with the germinal epithelium with epithelial cells (but without germ cells) was observed. Also, lobular-like structures delineated by a basement membrane within oil droplets, immune, and endocrine cells were observed with male gametes in some stage of development (Fig. 4).

**Figure 5:**
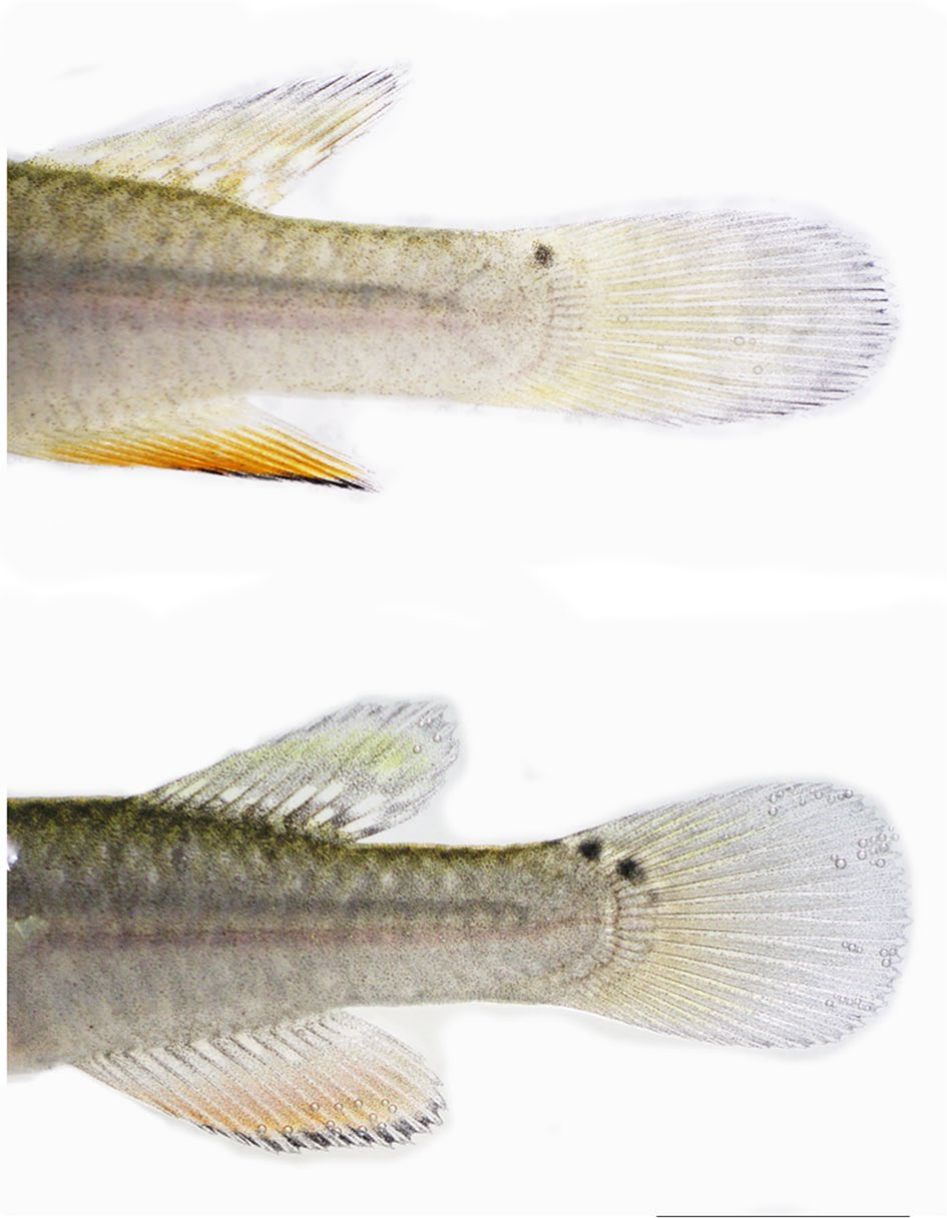
Phenotypic intersex in two *M. robustus* individuals at 53 ± 10 days after the beginning of the experiment. Male-typical colour pattern in the anal fin and female-typical one to three ocelli arranged at the base of the anal fin are apparent. Scale bar = 10 mm.

**Figure 6:**
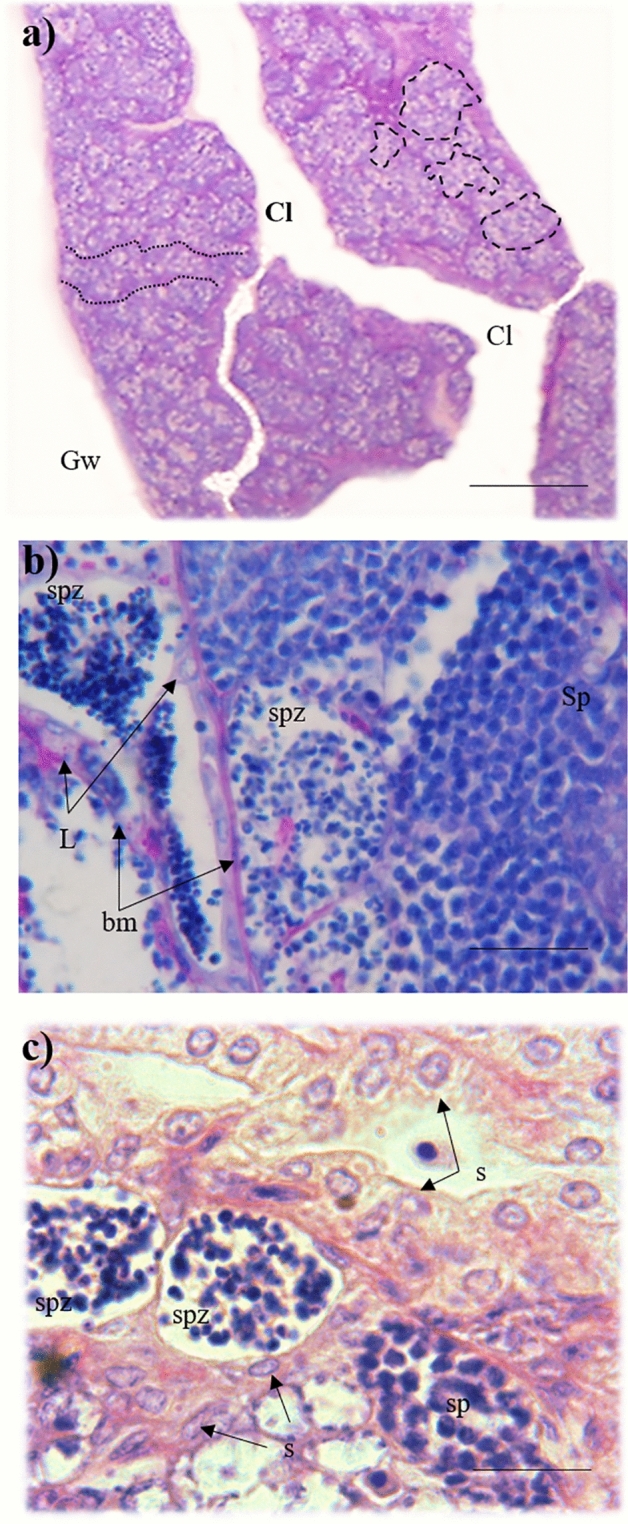
Gonad with male morphology at 53 ± 10 days after the beginning of the experiment. (**a**) Panoramic longitudinal section of a full testis of the lobular type with cysts (broken lines) within the lobules (dotted lines), with male reproductive cells in all stages of spermatogenesis. Central lumen (Cl) among the lamellas with male cells, projected from the wall of the gonad (Gw) can also be observed. Scale bar = 100 µm, [PAS staining]. (**b**) Detail of longitudinal section with Leydig cells (L) arranged between the basement membranes (bm) of lobules that contain cysts with primary spermatozoa (Sp) and mature spermatozoa (spz). Scale bar = 45 µm, [PAS staining]. (**c**) Detail of cells with acidophilic affinity in the cytoplasm, probably Sertoli cells (S), forming structures similar to testis ducts, adjacent to lobes with primary spermatocytes (Sp) and mature spermatozoa (spz). Scale bar = 15 µm, [HE staining].

After Day 53, the reproductive behaviour of sex changing fish included sporadic sigmoid displays (1.93 ± 0.28 per min). At this stage, the typical male colour pattern of three-tone orange bars was visible in the anal fin of sex-changing individuals, coexisting with the typical female colouration represented by small ocelli at the base of the caudal peduncle (Fig. 5). The gonads of these fish were composed of full testis of the lobular type (with cysts in the lobules containing male germ cells in all stages of spermatogenesis) but also ovarian lumen-like residues (Fig. 6a). Leydig cells were observed between the basement membranes of lobules containing cysts with primary spermatozoa and spermatozoa (Fig. 6b). The presence of cells with acidophilic affinity in the cytoplasm was observed, forming structures similar to ducts (Fig. 6c).

Finally, at Day 75, sex-changing fish started to exhibit exclusively male mating behaviour—“diving” (1.62 ± 0.13 per min) and “spawning” (1.13 ± 0.13 per min). This demonstrated that they had successfully courted and spawned with females in their compartment. At this time point, female harassment, attacks, operculum expansion and frontal displays occurred with minimal frequency in replicates with sex change but remained common in non-sex change replicates (Fig. 2). The ocelli in the caudal peduncle of sex changing fish gradually reduced their size until they disappeared. The gonads at this time point showed the same characteristics as at Day 53.

At the end of the experiment, three remaining sex-changed individuals were housed together with a female and confirmed to produce viable developing embryos.

## Discussion

We documented the first evidence of sequential hermaphroditism in an annual killifish and experimentally demonstrated how a competitive social environment triggers protogynous sex change. We then characterized gradual changes in behaviour, colouration and gonad structure during the sex change process.

The evolution of protogynous hermaphroditism is generally favoured when male reproductive output, as a function of specific age and size, increases faster than female reproductive output^9^. This happens when large males win agonistic encounters over the best territories and can monopolize food resources, habitat patches with lower mortality, reproductive resources and access to females^39^. In this context, it has been documented that the larger males of *M. robustus* always win male-male agonistic interactions with asymmetric body size, regardless of their age^40^. Although there is no information on potential male territoriality in *M. robustus*, dominant male annual killifish have superior access to females^30,41^ and are actively preferred by females^42^. Protandrous (male to female) sex change was not observed in our study. It is predicted that protandry evolves in monogamous mating systems, where mating is not size-selective (such as in group spawners), or when female fecundity largely increases with age^1,9^.

Sex change always occurred in only one individual per experimental unit. Unfortunately, body size and social hierarchy were not measured in our experiment (fish were too small to be individually marked) so we could not distinguish whether it was the largest female which changed the sex. Differential fitness of individuals through natural or sexual selection predicts which individual should change sex^3,11,43^. The original size-advantage hypothesis predicts that sex change is favoured when an individual reproduces most efficiently as one sex when young or small, but as the opposite sex when old or large^43,44^. When large males can monopolize matings with many females, protogyny is favoured and the largest females are recruited to the sex change trajectory, especially when access to males becomes limiting.

Ecological conditions and demographic parameters adjust individual reproductive investment and success, sometimes leading to a reversal of which sex is more competitive in sexual selection^1,45^. Our results reveal that sex change in *M. robustus* is related to competition among females to mate with males. In this context, we identify ecological scenarios which promote such competition among females in the temporary pools inhabited by *M. robustus* and stimulate protogynous sex change. In natural annual killifish populations, males may become scarce later in the season^29,30^ due to either sex-specific predation on colourful males with elaborate courtship behaviour^26,46^, mortality arising from male-male aggression^47^ or their combination^28^. While the more conspicuous colouration and more active behaviour of male *M. robustus* reflect the sex differences typical of annual killifishes, the relative scarcity of males in natural populations of *M. robustus*, and its potential seasonal dynamics, is currently not known. Annual killifish colonize new habitats through the dispersal of juvenile or adult fish^48^. In addition to the seasonal decline of males, sex change potentially supports reproduction in extremely small founding populations.

Asynchronous hatching (which can last up to three months^37^ or dispersal between populations^48^ may also affect the sex and size distribution of a population, with consequences for sex-specific benefits of individual strategy. In this context, Domínguez-Castanedo^49^ showed that *M. robustus* females started to choose large males under perceived mate competition risk, despite initially not displaying any male preference. Under female competition scenarios, choosy females increased their reproductive effort^49^. The previous scenarios of competition for mates were tested using focal females with free access to a male, with a female audience to produce perceived competition risk. In the current study, we tested females without the access to males, and they responded by changing sex.

This demonstrates that the life-history of *M. robustus* females is plastic and sensitive to social context, responding adaptively to limitations in access to males. Adaptive changes include selection of the best-quality males, increase in reproductive effort^49^ and, under certain circumstances, sex change. Within the order Cyprinodontiformes, protogyny is rare but confirmed in the swordtail *X. helleri* (Poecilidae)^14^. There are anecdotal reports of protogynous sex change in two African species of egg-laying killifish (family Nothobranchiidae)—non-annual *Aphyosemion striatum* and *Fundulopanchax amieti*, a species from a group of facultatively annual killifishes (T. Litz, personal communication). Huber^15^ reported a case of putative protogynous sex change in a wild-caught non-annual killifish *Aaptichilichthys websteri* (Procatopodidae) from an aquarium breeder when one of two females changed her sex to a functional male (and produced offspring) in captivity, after the original male died. Cauvet^16^ described a similar case in a captive group of 4 females and 1 male *Pseudepiplatys annulatus* (Nothobranchiidae) imported from the wild. However, the gonad histology of these individuals was not examined. Rivulid fishes also include the mangrove rivulus *Kryptolebias marmoratus* species complex which represents the only vertebrate lineage known to be a simultaneous hermaphrodite (i.e. simultaneous presence of male and female germ cells) capable of self-fertilization^50,51^. This suggests that functional sex is a relatively labile trait in cyprinodontiform fishes (including Rivulidae) and selection may act on propensity to adaptively adjust sex according to social and ecological conditions.

At the behavioural level, we cautiously suggest that male presence created a competitive social environment, and that this is a necessary condition to initiate sex change. This corresponds to the social conditions present in the experimental compartments with groups of females, where sex change occurred in 9 of 15 replicates. In contrast, the same level of perceived competition for access to males expressed by visual and chemical stimuli, but with no direct female-female behavioural interactions (i.e. in the compartments with isolated females), never resulted in sex change. Female *M. robustus* are highly aggressive to each other and body size does not play a role in predicting the winner. When males are not present, similarly-sized female avoid overt mutual aggression^40^, apparently to reduce the risk of injuries. In contrast, we commonly observed aggression between similarly sized females in this study.

Further evidence for the need for female-female competition in addition to access to male cues comes from our pilot studies, when we tested various experimental designs to induce sex change in *M. robustus* after it appeared to occur in natural populations. We tested sex change in isolated females as well as in small and large groups of females, and varied their size distribution, but we never recorded sex change in any of those experimental designs. Some females started to perform male reproductive behaviour, including (rare) apparent spawning, but although development was initiated in the eggs laid in those treatments the embryos never developed beyond a few days. This strengthens our suggestion that visual or olfactory male cues, in addition to physical interactions among females, are critical for the initiation of protogyny in *M. robustus*.

The mechanism of protogynous sex change through aggressive behaviour involves lowering the activity of brain aromatase, a key enzyme on oestrogen production. An immediate consequence of aromatase reduction is an increase in testosterone levels, particularly 11-ketotestosterone (the key male androgen in teleost fishes), affecting the brain, behaviour and morphology of sex changing individuals^52^. In vertebrate ovaries, the occurrence of survival factors for ovarian follicles (e.g. the estrogen 17β-estradiol) determines the start of follicular atresia. In the absence or deficiency of oestrogens, follicle cell death is activated^53,54^. Follicular integrity is related to high concentrations of oestrogens in follicular cells, which allows more significant expression of aromatase mRNA and gonadotropin receptors^55^. Our results demonstrated that the ovaries began their transition to testes before the expression of any behavioural or phenotypic traits. This suggests that a substantial decrease in the production of oestrogens, probably induced by aggression or dominance, triggers atresia of the ovarian follicles and subsequent increase in the synthesis of 11-ketotestosterone modulates changes in behaviour and colouration^56^. However, more research is needed in this species to determine timing of estradiol decrease and 11-ketotestosterone increase in relation to behavioural or colour changes.

Our histological observations revealed that the follicular atresia associated with sex change differs from atresia during the “normal” reproductive cycle of *M. robustus*^57^. The main difference consists in that the basement membrane does not break and the typical posterior intra-follicle vascularization of atretic ovarian follicles is therefore not present. This suggests that the basement membrane may be reused by the structure of the testis lobules, explaining why it remains integral over the sex change. Also, the testes of the secondary males retained characteristics of their previous female phase (e.g. ovarian-like lumen) documenting that the sex of a particular individual changed from a functional female to a male. This classifies *M. robustus* as a diandric species (with morphologically distinct primary and secondary males), showing two distinct pathways of sexual development in males^58^.

In this study, we demonstrated the first case of a sequential (protogynous) hermaphrodite in an annual killifish. We revealed that sex change is induced by the physical competition among females for a mate. Aggression among females could induce a decrease in oestrogen production, triggering a sequence of events from ovarian follicle cell death and consequent histological changes in the gonads, to the expression of male behaviour and colouration. We highlight the potential adaptive value of sex change in annual fishes despite their short lives.

## Methods

We used 240 *Millerichthys robustus* (120 males and 120 females) in this study. They were F4 generation individuals of fish originally collected in 2015 from a *M. robustus* population near of Tlacotalpan (N 18° 37′ 06.08″, W 95° 38′ 08.71″), Veracruz, México, under the authorization SGPA/DGVS/02404/14 of the Subsecretaría de Gestión para la Protección Ambiental, Dirección General de Vida Silvestre of SEMARNAT. *M. robustus* become sexually mature at the age of 3–4 weeks in the wild. Experimental fish were 8 weeks old at the start of the experimental period, i.e. at least four weeks after reaching sexual maturity.

### Experimental set-up

**Figure 7:**
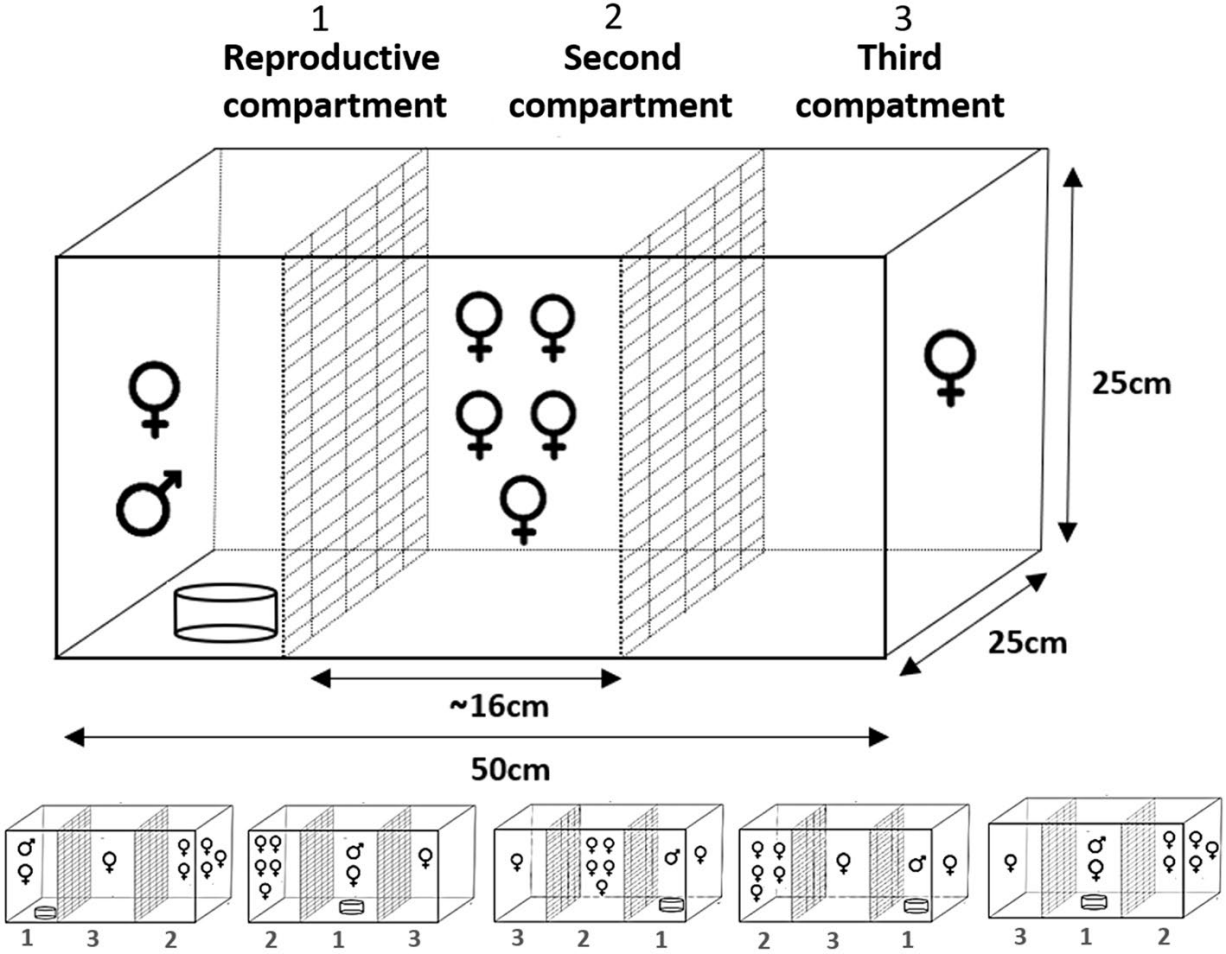
Experimental set-up to induce sex change in *M. robustus* through competition for access to mates. Mesh dividers create 3 compartments: (1) Reproductive compartment = mature male and female stimulus; (2) group of 5 females; (3) isolated female. The reproductive compartment included a glass container filled with peat as a substrate for spawning. Five remaining possible configurations of the three compartments are shown below.

We tested the role of mate competition to induce sex change in *M. robustus*. Our set-up was based on previous experimental work and pilot studies. It consisted of 15 experimental tanks (replicates), which were visually separated from each other. Each experimental tank was divided into three experimental compartments of 16 × 25 × 25 cm by two mesh screens (mesh size 3 mm) (Fig. 7). The mesh screen allowed visual and chemical interaction between fish in the different compartments but prevented physical contact.

To test protogynous (female-to-male) sex change, one compartment (reproduction compartment) contained a mature male and female. Males measured (mean ± standard deviation) 38.7 ± 10.4 mm standard length and females 34.4 ± 9.4 mm. This reproduction compartment was not experimental but generated a stimulus of the presence of an active male in the experimental tank. The male was physically isolated from the females in the other two compartments, but its presence could have incited competition for access to a male partner. The reproduction compartment included a glass container (8 cm deep, diameter of 10 cm) filled with 5 cm of peat that served as a substrate for spawning. The second compartment (audience compartment with female-female physical interactions) contained five mature females (35.2 ± 9.1 mm standard length). These females had no physical access to the male but were allowed to freely interact with other females in their compartment. In the third compartment (audience compartment without physical interactions), a single mature female (37.0 ± 10.3 mm standard length) was housed, with no physical access to any other fish (Fig. 7). The position of the three compartments was shuffled to control for the effect of the compartment arrangement. We used all six possible compartment arrangements (i.e., 1–2–3; 1–3–2; 2–1–3; 3–2–1; 2–3–1; and 3–1–2) in two groups of six experimental tanks (i.e. total of 12 experimental tanks). The remaining three experimental tanks were arranged randomly.

To test protandrous (male-to-female) sex change, we used the same experimental design as described for protogynous sex change, but replacing males for females (and vice versa) in the experimental compartments. In this test, males measured 33.8 ± 15.7 mm standard length and females 36.2 ± 8.8 mm. The protogynous and protandrous sex change experiments were not conducted simultaneously and different fish were used in each experiment. The experimental tanks were kept at 26 °C, pH 7.5 and Gh = 35 mg L^−1^, with a photoperiod of 14:10 h (L:D) throughout the experimental period. Fish were fed twice per day ad libitum with *Artemia franciscana* nauplii. A quarter of the water was changed weekly in all the experimental units.

Sex change was studied at behavioural, phenotypic (body colouration) and histological levels. Based on pilot observations, we set the experimental period to 90 days. Behaviour was recorded every second day from the beginning of the experiment until the first expression of behavioural units corresponding to the opposite sex. After the first expression of opposite-sex behaviour, we recorded the behaviour of that phenotypically intersexual individual during the sex change and once the sex change process was complete. In the replicates with no sex change, we observed randomly chosen individuals. This yielded a set of behavioural observations at days 1–33 (every second day, randomly selected individuals) and then more sparsely conducted observations at days 35–90 (irregular intervals of 1–2 weeks). Each observation lasted 20 min. We recorded the expression of 8 behaviours (Table 1) which were defined from previously published ethograms for courtship, reproduction and agonistic encounters of *M. robustus*^40,59^. They were composed of aggressive and courtship displays characteristic of female and male behaviour. We recorded the presence of any colour component of the opposite sex phenotype in fish from compartments 2 and 3 based on the sex-specific colouration in this species described by Domínguez-Castanedo et al.^36^.

For histological analysis, we sampled gonads at three time points—(i) when a behaviour typical of the opposite sex was first recorded (approx. 53 days after start of the experiment), (ii) when mixed expression of male and female colour phenotypes characters was observed (approx. 75 days), and (iii) when the behaviour and colouration of the opposite sex was attained (90 days). To minimize interference with the behavioural aspect of our experiment, we sacrificed two individuals at each of those time points. Individuals with transient (i.e. sex changing) phenotypes (behaviour, colouration) were always sampled. Fish were euthanized with an overdose of MS222 followed by posterior brain puncture. The gonads were extracted, fixed in 4% formaldehyde solution for 24 h and stored in 70% alcohol until histological preparation. Gonads were dehydrated in increasing concentrations of alcohol (80, 95, and 100%), cleared with xylene and embedded in Paraplast© paraffin with a melting point of 56 °C. Gonads were then cut to 6 μm slices and dyed with hematoxylin–eosin (HE), and periodic acid Schiff (PAS) reaction following Aguilar-Morales et al.^60^. The histological description of the gonads was based on^61–63^ Grier et al.^61^, Domínguez-Castanedo and Uribe^62^, and Domínguez-Castanedo et al.^63^.

Before the start of the experiment, we individually verified the reproductive competence of all females and males through the production of viable eggs (or capacity to fertilize them), respectively. After the sex change, we repeated this test with the fish that changed sex and remained alive after sampling for the histological analyses (n = 3). The mating partners used for these tests were fish not used in the experimental design.

### Data analysis

A total of 30 experimental replicates were completed (15 replicates for protogynous and 15 for protandrous sex change). Temporal dynamics in the expression of particular behaviours were tested in protogynous replicates. We compared the expression of 4 behaviours which are expressed by both sexes (and hence presented from the start of data recording) over the 90 days of the experiment. The response variables represented counts of behavioural acts per 20 min of observation and were initially modelled using Generalized Linear Mixed Models with a Poisson error distribution. To account for a large proportion of zero counts in some variables, we used a negative binomial error distribution with relaxed (linear) (*nbinom1*) and quadratic (*nbinom2*) parametrization. Fixed factors were “days from start of the experiment” (continuous) and occurrence of sex change (two levels: yes, no). Interaction between the two fixed factors was of central interest, as it indicated whether the expression of the particular behaviour changed in response to sex change (that occurred at approximately half of the experimental period). To account for non-independence of observations, tank identity was added as a random intercept to all models. Models with alternative parametrization (Poisson, nbinom1, nbinom2) were compared using the Akaike Information Criterion, the best fitting models were selected, and their assumptions (distribution of residuals, model misspecification) were checked. Behaviours that were observed only in the second part of the experiment were compared between replicates with and without sex change using Generalized Linear Mixed Models with one fixed factor (sex change, two levels: yes, no) for the period between 35 and 90 days, with otherwise identical model structure. To precisely test whether initial behaviour predicted the occurrence of sex change, we also compared female behaviour (until day 23) between replicates with and without subsequent sex change. We used Generalized Linear Mixed Models with Poisson and negative binomial error distribution, and with tank identity added as a random intercept.

All analyses were performed using the R statistical environment (v. 3.5.2)^64^ in the *glmmTMB*^65^ and *lme4* packages. Prior to formal statistical analysis, data variables were inspected for collinearity (using Variance Inflation Factor) and outliers. Model assumptions were checked using the *DHARMa* package^66^. The R code for data management and all analyses is available in the Figshare repository (https://doi.org/10.6084/m9.figshare.19363640).

### Approval for animal experiments

The use of animals was approved by the Subsecretaría de Gestión para la Protección Ambiental, Dirección General de Vida Silvestre of SEMARNAT (permit No. SGPA/DGVS/02404/14/15). All experiments were performed with accordance with relevant guidelines and regulations (individual experimental license CZ01285, and experimental procedures approval no. 31/2019 from the Ministry of Agriculture). Reporting in the manuscript follows the recommendations in the ARRIVE guidelines (https://arriveguidelines.org).

## Data Availability

All primary data and R code for statistical analyses are available on Figshare (https://doi.org/10.6084/m9.figshare.19363640).
